# Optimization of Pramipexole-Loaded In Situ Thermosensitive Intranasal Gel for Parkinson’s Disease

**DOI:** 10.3390/ph17020172

**Published:** 2024-01-29

**Authors:** Rushi Trivedi, Vahid Vikram Minglani, Ahmed M. El-Gazzar, Gaber El-Saber Batiha, Mohamed H. Mahmoud, Mitesh Patel, Meenakshi Patel

**Affiliations:** 1 Babaria Institute of Pharmacy, Varnama, Vadodara 391240, Gujarat, India; rushi29394@gmail.com; 2 Department of Pharmaceutics, School of Pharmacy, Parul University, Vadodara 391760, Gujarat, India; vaahiidminglani@gmail.com; 3 Department of Veterinary Forensic Medicine and Toxicology, Faculty of Veterinary Medicine, Alexandria University, Alexandria 5424041, Egypt; elgazzar@alexu.edu.eg; 4 Department of Experimental Pathology and Tumor Biology, Nagoya City University Graduate School of Medical Sciences, Nagoya 467-8601, Japan; 5 Department of Pharmacology and Therapeutics, Faculty of Veterinary Medicine, Damanhour University, Damanhour 22511, Egypt; dr_gaber_batiha@vetmed.dmu.edu.eg; 6 Department of Biochemistry, College of Science, King Saud University, Riyadh 11451, Saudi Arabia; mmahmoud2@ksu.edu.sa; 7 Research and Development Cell, Department of Biotechnology, Parul Institute of Applied Sciences, Parul University, Vadodara 391760, Gujarat, India; 8 Department of Pharmaceutics, School of Pharmacy, Faculty of Pharmacy and Research and Development Cell, Parul University, Vadodara 391760, Gujarat, India

**Keywords:** intranasal, in situ gel, Pramipexole dihydrochloride, anti-Parkinson, thermoreversible

## Abstract

The objective of the present work was to develop and optimize an intranasal in situ gel of Pramipexole dihydrochloride for enhanced drug delivery, better patient acceptability, and possible proper treatment of Parkinson’s disease. Preliminary studies were performed to select formulation components and identify key variables affecting the formulation. The optimization of the in situ gelling system of Pramipexole dihydrochloride was achieved by applying 3^2^ full factorial design using Design-Expert^®^ software (Stat-Ease 9.0.6 version) and taking concentrations of Poloxamer 407 (X_1_) and HPMC K4M (X_2_) as independent variables. The gelling temperature, gel strength, and percentage of drug diffused after 8 h were taken as dependent variables. The software provided an optimized formulation, with 16.50% of X_1_ and 0.2% of X_2_ with the highest desirability. An in vivo drug retention time study was performed for the optimized formulation in Wistar rats. The results of the optimization process demonstrated that the selected gel formulation exhibited desirable characteristics, including gelation near body temperature, good gel strength, suitable viscosity, and sustained drug release. The optimized formulation displayed significantly higher drug retention, lasting about 5 h, versus the plain poloxamer gel formulation. Hence, it was concluded that the optimized formulation will remain affixed at the site of application for a significant time after intranasal administration and consequently sustain the release of the drug. The optimized formulation was found to be stable during the stability studies. The developed dosage form may improve patient compliance, enhance nasal drug residence, and offer sustained drug release. However, further clinical studies are necessary to validate these findings.

## 1. Introduction

Parkinson’s disease (PD) is a chronic, progressive, age-related condition that is the most prevalent complicated neurodegenerative disorder after Alzheimer’s disease [1,2]. In this disease, there is a gradual loss of brain cells that make and store dopamine in the substantial nigra part of the brain [3]. Due to this loss, an inadequate amount of dopamine reaches the cortex and thalamus, affecting their functioning in controlling muscle movement, leading to abnormalities in PD patients [4]. The onset of this disease is initiated when free radical synthesis occurs in dopaminergic neurons, causing stress-induced oxidative neurodegeneration [5]. The characteristic clinical features of PD include bradykinesia, rigidity, and tremors [6]. Compared to other common neurological illnesses, the number of PD patients is rapidly growing globally. According to a recent statistical study, there are currently seven to ten million people suffering from this condition worldwide, and by the year 2050, that number is expected to exceed twelve million [7]. Treatment includes the direct supply of dopamine and its agonists to the brain or catechol O-methyltransferase inhibitors that lead to an increased half-life of dopamine [8]. Treatment also includes controlling symptoms with anticholinergics.

Pramipexole, classified as an aminothiazole dopamine agonist, has selective affinity to act upon the D2 subfamily of dopamine receptors [9]. However, Pramipexole is far more effective at stimulating postsynaptic dopamine D2 and D3 receptors in dopaminergic circuits where dopamine release is reduced due to presynaptic neuron degeneration. Pramipexole has been claimed to have advantageous effects against depression, a property likely due to pramipexole-induced activation of postsynaptic dopamine D2 receptors [10]. These dopamine agonists benefit from possessing a long half-life, leading to enhancement of the sense of a continued postsynaptic activation of the receptors, making it the first-line medication for early PD. The drug has reasonably good oral bioavailability, but nonetheless, 80% of the absorbed drug is cleared through the kidneys [11]. However, the availability of the drug in the brain is limited due to the presence of the blood–brain barrier (BBB). Intranasal drug delivery offers a promising alternative, enabling direct and rapid access to the central nervous system.

Studies suggest that olfactory regions do not have a tough lipid blood–brain barrier. Therefore, intranasal medication delivery might be viewed as advantageous in ensuring the drug’s immediate entrance into the brain. Intranasal administration has been a highly acknowledged form of therapy since ancient times. This method of drug administration has successfully been used to deliver many drugs prescribed to treat PD [12,13,14,15]. Hence, an endeavor was made to prepare the thermosensitive in situ intranasal gel consisting of Pramipexole for the effective treatment of PD. The dose of the chosen drug is very small (starting from 0.125 mg), so it can be easily accommodated by any formulation for the treatment route. However, the route’s drawback is the drug’s brief and decreased residence time, brought about by rapid mucociliary clearance at the site of administration. This issue can be solved by the in situ gelling method.

## 2. Results and Discussion

### 2.1. Preliminary Studies

The temperature at which gelation occurred in the Poloxamer 407 solutions of various concentrations was in decreasing order. The temperature required for gel formation dropped as the poloxamer concentration in the solution increased. When there is an increase in the concentration of Poloxamer 407, the hydrophobic portion of the polymer also increases simultaneously, leading to gelation at a lower temperature. The gelation temperature ranged from 39 ± 0.5 °C to 27 ± 0.5 °C for different concentrations of Poloxamer solutions from 16 to 21, respectively (Table 1). The 20% *w*/*v* solutions of Poloxamer 407 (with a gelation temperature of 29 ± 0.5 °C) were used to check the effect of the excipients to be added to the formulation. Upon the addition of the drug, Carbopol 934 and HPMC K15M, it was observed that the gelation temperature of the Poloxamer solution decreased. However, upon the addition of benzalkonium chloride and sodium chloride, the gelation temperature of the Poloxamer solution increased (Table 2). This occurs as a result of the effects of the excipients on the production of micelles, which alter the hydrogen bonds between the polymer blocks and the water molecules.

**Figure 1:**
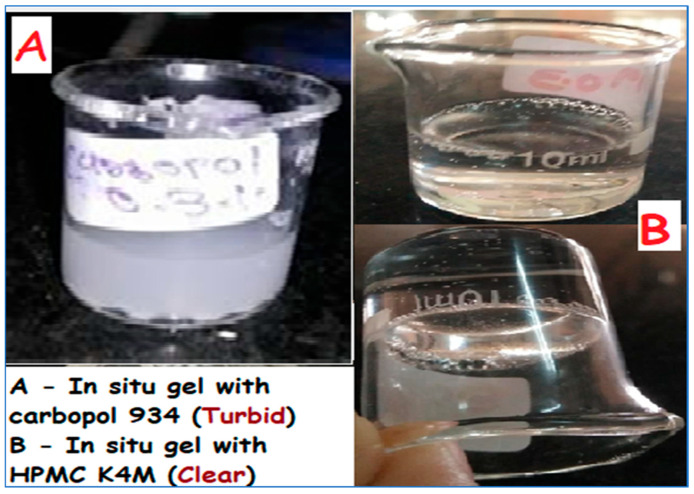
Effect of Carbopol 934 and HPMC K4M on in situ gel of Pramipexole dihydrochloride.

The study conducted for the screening of Carbopol 934 and HPMC K4M showed that the Pramipexole in situ gel made by adding Carbopol 934 turned white during storage (Figure 1), indicating the precipitation of the drug. Moreover, a Fourier transformed infrared (FTIR) spectroscopic scan of the drug and Carbopol indicated interaction between the two. Hence, it was determined to optimize the formulation using Poloxamer 407 and HPMC K4M.

### 2.2. Drug Excipient Interaction Study

**Figure 2:**
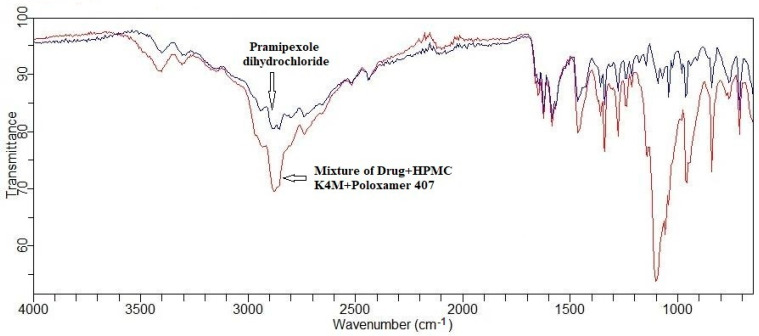
FTIR scan of drug and mixture of drug and polymer.

A FTIR scan was performed for a physical mixture consisting of the drug and polymer. The FTIR scan performed for Pramipexole dihydrochloride revealed recognizable peaks at 2737.7, 1584.1, 1094.0, 3401.2, 3304.3, and 2939.0 cm^−1^ representing a C-C aliphatic group, C=C stretching, C-N stretching, N-H stretching of the primary amino group, N-H stretching of the secondary amino group, and aromatic C-H stretching, respectively. The detection of all these peaks in the infrared spectra generated from the drug-polymer mix demonstrates that no discernible incompatibility between the drug and the polymers exists (Figure 2).

### 2.3. Experimental Design

The thermoreversible intranasal gel of Pramipexole was finally optimized using Poloxamer 407 and HPMC K4M based on preliminary research and a literature review. The formulations were provided with an in situ gelling property by the incorporation of Poloxamer. Enhanced gel strength was provided by the addition of HPMC in the formulation. The factorial design batches were created in accordance with the recommendations of Design Expert software.

All the formulations prepared were transparent in the liquid as well as gel state, with a smooth texture. Nasal formulations should have a pH between 4.5 and 6.5, and the pH of all the developed batches was found to be in the range of 4.79–5.22, indicating that the formulations are unlikely to trigger any irritation to the nasal mucosa and tissues. The viscosity at 4 °C was found to be in the range of 17 ± 3.3 cps to 45 ± 2.28 cps, and at 37 °C, it was in the range of 4965 ± 6.12 cps to 5938 ± 3.14 cps. The force of mucoadhesion was found to be in the range of 1632.09 ± 10.5 dyne/cm^2^ to 3862.5 ± 14.52 dyne/cm^2^. Table 3 presents the results of the evaluation parameters of the developed formulations. The overall observation was that formulation PG9 had better viscosity and high mucoadhesive force, hence strength, which is evidently caused by the formulation’s high independent variable concentration.

Gelling temperature in all the prepared formulations was found to be in the range of 30–37 °C, indicating that the independent variables had a substantial impact on the dependent variables. The range of gel strength was 30 to 47 s, and the % drug diffused after 8 h was in the range of 85.52% to 95.65%, as displayed in Table 3. The statistical analysis of the obtained results was performed using Design Expert software. Table 4 summarizes the analysis of variance for response parameters for the 3^2^ full factorial design of the in situ gel. The significance (*p* < 0.05) of the ratio of mean square variation, being an attribute of the regression coefficient, was examined by the implementation of analysis of variance (ANOVA). The residual error was additionally tested, and the model’s importance was established. Results for the response parameters revealed that the gelling temperature, gel strength, and % drug diffused after 8 h were all statistically significant (*p* < 0.0500). This indicates the significance of incorporating the corresponding model terms in the statistical analysis. After observation of the *p* value for the response parameters, the *p* value for the gelling temperature, gel strength, and % drug diffused after 8 h were 0.0036, 0.0029, and 0.0033, respectively. Additionally, the quadratic model was shown to be of significance for the gelling temperature, gel strength, and percent of drug diffused after 8 h.

### 2.4. Gelling Temperature

The gelling temperature appears to be within the range of 32 to 35 °C. R^2^ was determined to be equal to 0.9896. The resultant F-value was 57, indicating that the model is found to be significant. There is only a 0.36% chance that an F-value this large could be caused by noise. “Adeq Precision” determines the signal to noise ratio. When the ratio is higher than 4, it is regarded as preferable, and the ratio found for the present model was 22.183, implying that the signal is adequate. The current model can thus be utilized to navigate through the design space. The other information obtained from the ANOVA table was regarding the variables. The *p* value for the factors X_1_ and X_2_ was calculated and found to be 0.003 and 0.01, respectively, implying that X_1_ and X_2_ have significance in the model. For model analysis, a quadratic model can be used to express the result of the analysis. The following is the fitted equation for the responses:

Gelling temperature °C (Y_1_) = +32.67 − 0.83 X_1_ − 2.33 X_2_ + 0.75 X_1_X_2_ + 0.50 X_1_^2^ − 0.01X_2_^2^

**Figure 3:**
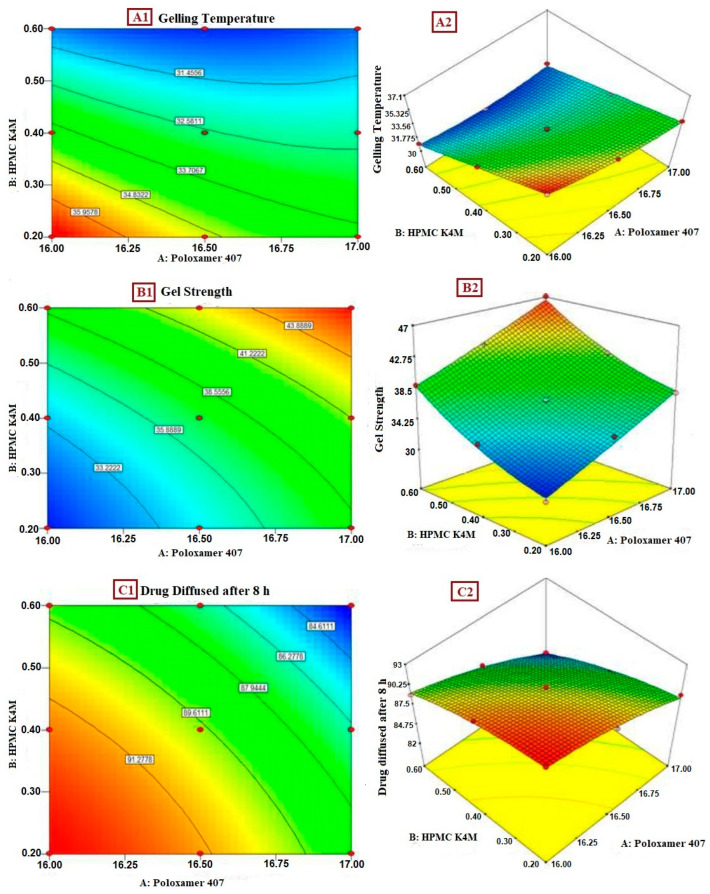
(**A1**) Contour plot of gelling temperature; (**A2**) 3D response surface plot of effect of polymer on gelling temperature; (**B1**) contour plot of gel strength; (**B2**) 3D response surface plot of effect of polymer on gel strength; (**C1**) contour plot of drug diffused after 8 h; (**C2**) 3D response surface plot of effect of polymer on drug diffused after 8 h.

By observing the above equation, it is evident that both of the factors, i.e., the amount of Poloxamer 407 (X_1_) and HPMC K4M (X_2_), tend to have a negative effect on the gelling temperature of the formulated in situ gel. This signifies that as the amount of both variables increases, the gelling temperature decreases. A graphical representation of the main effects and interaction effects of the independent components is provided in Figure 3(A1,A2), along with a contour plot and three-dimensional surface response graphs for the gelling temperature.

### 2.5. Gel Strength

The range of the gel strength was calculated to be within 25 to 50 s. R^2^ was found to be 0.991. The F-value was calculated, and it was found to be 65.85, denoting the model as significant. There is only a 0.29% chance for an F-value this enormous to be caused due to noise. Additional details about the variables were discovered from the ANOVA table. The *p* value for factors X_1_ and X_2_ was discovered to be 0.002 and 0.001, respectively, indicating that X_1_ and X_2_ have significance in the current model. The interaction effect, as well as the quadratic effect of X_1_ and X_2_, were found to be significant. For model analysis, a quadratic model can be used to express the outcome. The fitted equation for the responses is as follows:

Gel Strength (Y_2_) = +37.22 + 3.83X_1_ + 4.17X_2_ + 0.000X_1_X_2_ + 0.17X_1_^2^ + 1.17X_2_^2^

The aforementioned equation evidently signifies that each of the factors, the amount of Poloxamer 407 (X_1_) and of HPMC K4M (X_2_), tends to have a positive impact on the gel strength of the formulated in situ gel. This signifies that as the amount of both the variables increases, the gel strength increases. The contour plot and three-dimensional response surface graphs for gel strength are provided in Figure 3(B1) and Figure 3(B2), respectively.

### 2.6. % Drug Diffused after 8 h

The drug diffused after 8 h was found to be in the range of 85 to 100%. The value of R^2^ was found to be 0.9901. The estimated F-value was found to be 60.26, implying that the model is significant. There is just a 0.33% chance that an F-value this high could have been caused by noise. “Adeq Precision” is a method to determine the signal to noise ratio. The desirable ratio is usually considered to be greater than 4, and the ratio found for the present model was 24.05, signifying that the signal is adequate. This model is functionally appropriate to navigate the design space. The other information obtained from the ANOVA table was regarding the variables. The *p* value for the factors X_1_ and X_2_ was calculated to be 0.001 and 0.001, respectively, pointing to X_1_ and X_2_ having significance in the model. As the response, drug release after 8 h followed the quadratic model, with no interactions noted between the independent factors, and a quadratic effect observed. The result obtained may be utilized for model analysis as a quadratic model. The fitted equation aimed for the responses is given below:

% Drug release after 8 h (Y_3_) = +91.89 − 2.83X_1_ − 2.00X_2_ − 0.25X_1_X_2_ − 0.83X_1_^2^ − 0.33X_2_^2^

By examining the equation above, it is evidently clear that both of the factors, the amount of Poloxamer 407 (X_1_) and HPMC K4M (X_2_), tend to have a positive influence on the drug diffusion of the in situ gel formulation, signifying that as the amount of both the variables decreases, the drug release increases. The contour plot and three-dimensional response surface graphs for the drug diffused after 8 h are provided in Figure 3(C1) and Figure 3(C2), respectively.

### 2.7. Validation of Model

As per suggestions of the Design Expert software, three additional formulations were created to ascertain and validate the trustworthiness of the mathematical models built by implementing full factorial design. The three checkpoint batches, PG10, PG11, PG12, were formulated with the level of X_1_ and X_2_ as 16.31 and 0.48, 16.18 and 0.32, and 16.45 and 0.22, respectively, while maintaining the same amount for each of the other components in the formulation. Table 5 displays the actual and predicted responses with a percent bias less than 5%, implying that the experimental results are compatible with the expected values, confirming the predictability and validity of model.

### 2.8. Optimization of Formulation by Graphical Method

**Figure 4:**
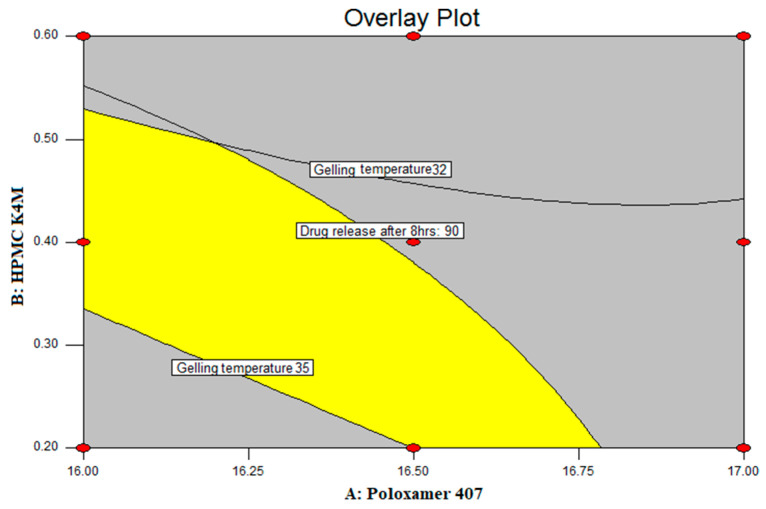
Overlay plot.

The optimized batch was chosen by applying constraints on gelling temperature (in the range of 34–37 °C), gel strength (maximum), and% drug diffused after 8 h (maximum). The overlay plot, which reflects the yellow region shown in Figure 4, represents the area of the experimental region. According to the Design Expert software, the PG4 formulation, with a concentration of the polymer of Poloxamer 407 (X_1_) and HPMC K4M (X_2_) of 16.50% *w*/*v* and 0.2% *w*/*v*, respectively, tends to be highly desirable. Therefore, this batch was considered as the optimized batch.

**Figure 5:**
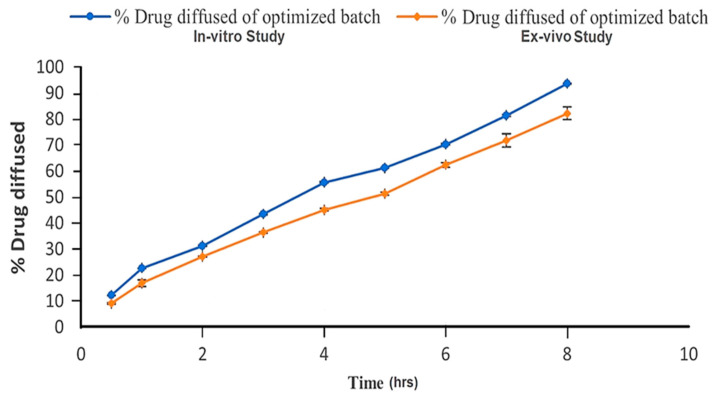
In vitro and ex vivo drug diffusion profile of optimized formulation (PG4).

The prepared optimized formulation was evaluated for an ex vivo drug diffusion study. It showed 82.17% cumulative drug diffusion through goat nasal mucosa after 8 h. A graphical representation of the comparison between the in vitro drug diffusion study and the drug diffusion from optimized formulation PG4 through goat nasal mucosa is shown in Figure 5. It is evident from the graph that the release through the goat mucosa is slower than that in the in vitro drug release study. The in vitro drug diffused data of the optimized formulation were evaluated kinetically using various mathematical models, such as the zero-order, first-order, Higuchi, and Hixon–Crowell models. The formulation followed zero-order release kinetics, with a regression value of 0.9969, indicating that the mechanism of release was non-Fickian diffusion-regulated. The next closest kinetic model followed by the optimized formulation was the Hixon–Crowell model, with an R^2^ value equal to 0.982, indicating the formulation follows the erosion mechanism for drug release.

### 2.9. In Vivo Drug Retention Time

**Figure 6:**
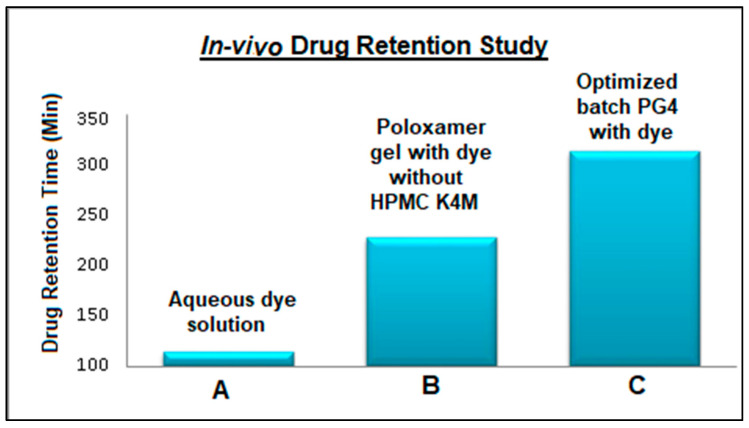
In vivo drug retention times of intranasal Pramipexole dihydrochloride gel.

The resultant in vivo drug retention time found for the optimized nasal in situ gel formulation (batch PG4) is shown in Figure 6. The presence of dye in the nasal tissue of the rat was confirmed by taking samples at regular intervals. As the dye appeared on the swab, it indicated the presence of the gel at the site of administration. Plain Poloxamer gel showed a significant drug retention time compared to the control (aqueous dye solution). The formulation containing mucoadhesive polymers displayed significantly higher drug retention for about 5 h versus the plain Poloxamer gel. This study proved that the retention time of the in situ gelling system increases with the addition of HPMC K4M.

### 2.10. Stability Studies

There were no significant differences found in the physical appearance, pH, drug content, percentage of drug diffusion, or gelling characteristics before, during, or after the stability study period. The formulation appeared clear and transparent throughout the stability study period. No drug precipitation was observed. Nasal mucosal pH is slightly acidic; the initial (0 day) pH of the optimized formulation was 5.18. Similar pH values were found during and after the stability study of the formulation. The results of viscosity on day zero, the viscosity at 4 °C and at 25 °C, drug content uniformity, and mucoadhesive force were 24 ± 3.21, 5785 ± 3.14, 95 ± 1.1, and 3082.09 ± 15.3, respectively. The variation in the results during the entire duration of the stability study was less than 5%. Hence, it was concluded that the optimized in situ gel formulation of Pramipexole dihydrochloride was stable for 90 days. The graphs obtained for the in vitro release study were also overlapping, indicating the stability of the formulation during the complete study period.

## 3. Materials and Methods

### 3.1. Materials

Pramipexole dihydrochloride was received as a gift sample from Sun Pharma, Vadodara, Gujarat, India. Poloxamer 407 was procured from Sigma Life Sciences, Mumbai, India, and hydroxyl propyl methyl cellulose (HPMC) K4M was procured from Chemdyes Corporation, Vadodara, Gujarat, India. Benzalkonium chloride and sodium chloride were procured from Suvidhinath Laboratories, Vadodara, Gujarat, India.

### 3.2. Methods

#### 3.2.1. Formulation of Thermoreversible In Situ Gels

Preparation of thermoreversible in situ gelling systems was conducted via the “cold method” by utilizing Poloxamer 407 and HPMC K4M as gelling agents [16,17]. Poloxamer 407 was dissolved in deionized distilled water at room temperature and then kept overnight at 4 °C to ensure complete solubilization. A separate solution of HPMC K4M was also prepared after its solubilization in water. Both solutions were then mixed, and other excipients were added. Finally, the drug was added with continuous agitation in the above mixture, and the solution was stored overnight at 4 °C. To adjust the final volume, ice-cold distilled water was added. The liquid was then cooled until it was ready for use [18,19,20].

#### 3.2.2. Preliminary Work

Calculating the quantity of drug added to the formulation, the minimum dosage of 0.125 mg of medication was taken into consideration. One drop of this drug concentration needs to be placed inside the nostril. While performing this, it was found that 1 mL of solution contains 20 drops. As a result, the dosage was calculated appropriately, and 25 mg of the drug was introduced into the 10 mL in situ gelling formulation.

Poloxamer 407 is a polymer consisting of a triblock of hydrophilic and hydrophobic poly(ethylene oxide) and poly(propylene oxide) structures [21]. At low temperatures, this polymer stays in a liquid state owing to hydrogen bonding occurring between the polymer monomers and molecules of water [22]. As the temperature increases, the hydrogen bonding ceases to exist, and hydrophobic interactions convert the solution into a gel [16].

To check the gelation property of Poloxamer 407, solutions of various concentrations (ranging from 16 to 21% *w*/*v*) were created, and the temperatures at which they gelled were noted. All the excipients effecting the gelation temperature of Poloxamer 407 solutions were also assessed individually. To provide the final formulation of the mucoadhesive characteristics and good gel strength, Carbopol 934 and HPMC K4M were investigated during the preliminary investigation.

#### 3.2.3. Drug Excipient Compatibility Studies

FTIR spectra for a mixture of the drug and excipients as well as the drug in its pure form were recorded using a FTIR spectrophotometer (Cary 630 FTIR Spectrometer, Agilent technologies). The interpretation of the IR scan showed the FTIR spectra of (A) Pramipexole dihydrochloride and (B) the physical drug and excipient mixture.

#### 3.2.4. Experimental Design—Two-Factor and Three-Level Factorial Design

The application of experimental designs has gained noticeable popularity for developing and optimizing formulations [23,24,25]. To optimize the nasal in situ gel containing Pramipexole dihydrochloride, a three-level and two-factor (3^2^) factorial design was employed [26]. Poloxamer 407 (X_1_) and HPMC K4M (X_2_) were chosen as the independent variables (factors), having three varying levels (low, middle, and high). Based on the results of the preliminary research, the concentration ranges for both variables were chosen. Gelation temperature (Y_1_), gel strength (Y_2_), and % drug diffusion after 8 h (Y_3_) were chosen as dependent variables (responses). The statistical experimental design was created and processed for optimization using Design Expert software. The design, including investigated factors and responses, is shown in Table 6. After applying the statistical design, the batch compositions that were created are listed in Table 7.

#### 3.2.5. Evaluation of In Situ Gels

##### Physicochemical Parameters

The color and texture of the prepared in situ gel were evaluated. The color was checked through visual inspection, and the formulation’s clarity was assessed on both white and black backgrounds.

##### pH of the Gels

A total of 10 mL of each formulation were extracted into in a 25 mL beaker, and the pH was recorded afterward. A solution of pH 4 and pH 8 was used to calibrate the pH meter (PM100, Welltronix, EIE Instruments Pvt. Ltd., Ahmedabad, Gujarat, India). After calibration with pure water, the pH was then measured batch by batch.

##### Drug Content

In a volumetric flask of 10 mL, 1 mL of the formulation was added. It was diluted using double-distilled water, and the volume was adjusted as per the designated mark. Ten milliliters of double-distilled water were used to further dilute one milliliter of this solution. To determine the drug concentration in the prepared dilution, the calibration curve of the drug in double-distilled water was prepared. The absorbance of the known concentrations of the drug was plotted on the graph to obtain the regression coefficient and the equation to find the unknown concentration of the drug. The calibration curve was prepared three times to obtain the value of standard deviation. The equation Y = mx + c was obtained from the graph, where Y is absorbance, m is the slope obtained in the graph, x is the drug concentration, and c is the intercept. The slope was found to be equal to 0.022, and the intercept was 0.006. The R^2^ was calculated as 0.999, indicating a high level of correlation. The Limit of Detection (LOD) and Limit of Quantitation (LOQ) values were found to be 1.02 and 3.08, respectively. A repeatability study was performed with 10 mcg/mL solution with six replicates. The mean, SD, and RSD values were found as 0.243, 0.002, and 0.888, respectively. The concentration of the medication was determined by measuring the absorbance of the produced solution at 262 nm using a UV visible spectrophotometer (Shimadzu UV-1800, Kyoto, Japan).

##### Gelling Temperature

The gelling temperature is the temperature at which a liquid phase turns into a gel. For thermoreversible nasal gel, a reasonably ideal gelation temperature would range from 30 to 36 °C. The gelation point was believed to be the temperature at which compositions would cease to flow when test tubes were tilted 90 degrees [27].

##### Viscosity of the Gels

The viscosity of the formulated batches was assessed using a Brookfield Viscometer (Brookfield, Pvt. Ltd., Model LVDV2P230, Toronto, OH, USA). The viscosity was measured at two contrasting temperatures (4 °C and 37 °C). The gel to be assessed was put in a tiny sample holder, and the spindle (helipath spindle S-96) was dropped into it perpendicularly and rotated at different speeds (10–100 rpm). The viscosity of the solid as well as gel states of the formulation was thus determined [18].

##### Gel Strength

Prepared in situ gels were placed in a 10 mL test tube and made to undergo gelation in a temperature-regulated water bath at 37 °C. A syringe piston was modified by sticking a load carrying cap on the pushing end. The piston was then lowered onto each gel one at a time, and a weight of 10 g was positioned over the attached cap. The gel strength was calculated by measuring the time (s) necessary to lower the needle through the gel [28].

##### Determination of Mucoadhesive Force

The amount of force required to separate the goat nasal tissue from the formulation served as an evaluation of the mucoadhesive force of formulation, using modified balance [28]. The left arm of the measuring balance was modified such that the side had a two-piece cylindrical glass vial. The lower piece was fixed with the base of balance, and the upper piece was attached with the hook of balance. With the mucosal side facing out, two pieces of goat nasal mucosa were immediately cut and fastened with rubber bands to the upper cylindrical component and lower glass vial. One milliliter of the formulation was applied to the nasal mucosa such that it was sandwiched between the upper and lower cylindrical pieces. The formulation was allowed to stick properly on both pieces of nasal mucosa. Small steel balls were then continuously added to the opposite side of balance until the two cylindrical parts separated from one another. The detachment stress or mucoadhesive force was estimated by calculating the nominal weight that was needed to separate both the vials. The following formula was utilized for calculating the weight needed for the detachment and to determine the mucoadhesive strength [29]:

Bioadhesive strength = mGA

where m = mass required for detachment, and G = acc. Due to gravity = 980 cm^2^/s, A = area to which the tissue is exposed = 0.785 cm^2^.

##### In Vitro Diffusion Studies

Studies on drug release were carried out by utilizing a vertical Franz diffusion cell (Orchid scientific, Nashik, India) [30]. The experiments were conducted by the utilization of a magnetic stirrer under continuous stirring at 200 rpm. The cellophane membrane was fastened to the donor chamber tube. A total of 20 mL of phosphate buffer with pH 5.8 was added in the receptor chamber, and the mixture was constantly stirred. The cell was equilibrated at 37 ± 2 °C. The drug formulation was placed on the dorsal surface of the cellophane membrane. One milliliter of receptor fluid was periodically taken out and replaced with new receptor fluid to keep the washbasin condition stable. The portion of the drug that permeated through the cellophane membrane was measured at a wavelength of 262 nm using a UV spectrophotometer.

##### Ex Vivo Diffusion Study

Fresh and meticulously extracted nasal mucosa from the nasal cavity of sheep from a local slaughterhouse was acquired [24]. The extracted nasal mucosa was submerged into a phosphate buffer solution at a pH of 5.8, and samples of the tissues were put on the vertical Franz diffusion cell shortly thereafter. The acceptor chamber was filled with pH 5.8 phosphate buffer solution at 37 °C. The donor chamber was filled with the Pramipexole dihydrochloride formulation. For a period of 8 h, 1 mL of samples were taken at predefined intervals from the acceptor compartment and replaced with phosphate buffer of pH 5.8 after each of the samples was withdrawn. The withdrawn samples underwent filtering and analysis. A UV-visible spectrophotometer was used to calculate the amount of drug that had permeated through the membrane.

##### In Vivo Drug Retention Time

**Figure 7:**
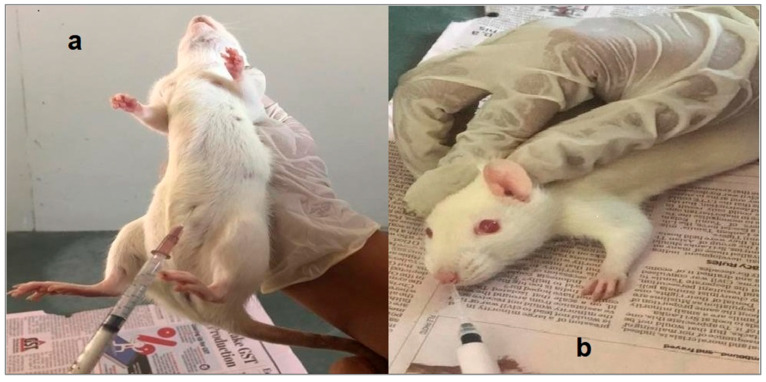
In-vivo drug retention time study: (**a**) inducing anesthesia, (**b**) injection of formulation in the nostril of rat.

All animal studies were conducted following institutional animal ethics committee approval of the protocol (BIP/IAEC/2018/10). Male Wistar rats were kept in individual metal cages inside the animal house of Babaria Institute of Pharmacy (Varnama, Vadodara, Gujarat, India) with the temperature maintained at 25 °C. All the animals were provided with standard diet and water ad libitum. Six male Wistar rats, having weights ranging from 250 to 320 g, were intraperitoneally injected with urethane solution (1200 mg/kg) to induce anesthesia. An optimized formulation of 5 µL Pramipexole dihydrochloride gel comprising the dye xylene cyanol (3 mg/mL) was injected into the nostril of one of the rats at a depth of 0.5 cm using a micropipette. Cotton-tipped applicators were used to swab the pharyngeal cavities of the rats that received the dose. The swab samples were taken every minute for the first 30 min and then at an interval of 1 h, 1½ h for next 8 h. The samples were visually analyzed. Rats were also given 5 µL of plain Poloxamer gel (lacking mucoadhesive polymer) and 5 µL of dye-loaded fluid (made in normal saline) as controls [31,32]. A pictorial presentation of the administration of anesthesia and the injection of the formulation in the nostril of an experimental subject is given in Figure 7.

#### 3.2.6. Stability Study

Stability studies of Pramipexole dihydrochloride nasal gel were performed at 4 ± 2 °C/55 ± 5% and 25 ± 2 °C/65% ± 5% temperature and relative humidity. The in situ gel was placed in bottles that were dry, airtight, moisture-proof, and clean. They were kept away from light. Samples of the gel were removed at intervals of 0, 15, 30, 60, and 90 days, and their appearance, pH, homogeneity of content, percentage of drug diffusion, and gelling characteristics were assessed for Pramipexole dihydrochloride content [33].

## 4. Conclusions

A nasal in situ gel of Pramipexole dihydrochloride was developed in the study reported here. The thermoreversible intranasal in situ gel was optimized by the implementation of 3^2^ full factorial design. Poloxamer 407 and HPMC K4M were employed as independent variables, and their effect on pH, gelling temperature, gel strength, and mucoadhesive force was observed, and in vitro and ex vivo release studies were performed. The formulation prepared by combining Poloxamer 407 and HPMC K4M in concentrations of 16.5% *w*/*v* and 0.2% *w*/*v*, Batch PG4, showed a desirable gelling temperature (35 °C), gel strength (35 s), and % drug diffused after 8 h (93.65%), proving that Poloxamer 407 and HPMC K4M, along with the drug used, provide sustained release of the drug. As the concentration of Poloxamer 407 and HPMC K4M was raised, it was noticed that the mucoadhesive force and gel strength of the formulation increased. During the in vivo drug retention time study, it was found that the formulation was retained in the rat nasal cavity for 5 h. Therefore, loss of the drug due to mucociliary clearance is not supposed to be due to the presence of mucoadhesive polymer. The optimized formulation (Batch PG4) excels in the short-term stability studies. Hence, it can be concluded that the optimized nasal in situ gel of Pramipexole dihydrochloride remained affixed to the site of application for a longer period of time. This improved the nasal residence time of the formulation and facilitated a controlled release of the drug. The resulting dosage form may potentially enable direct access to the brain through the olfactory region or trigeminal nerves, thereby enhancing the biological availability of the drug in the brain. This formulation will improve patient compliance by decreasing the dosing frequency, and it can be used as an alternative mode of delivery for the drug for the treatment of Parkinson’s disease. Nevertheless, additional clinical investigations are imperative to validate these results and unlock the complete therapeutic potential of this novel intranasal gel formulation.

## Figures and Tables

**Table 1:** Results of gelation temperature of Poloxamer 407 at different concentrations.

Concentration of Poloxamer 407 (% *w*/*v*)	Gelation Temperature (°C) (*N* = 3)
16	39 ± 0.5
17	37 ± 0.3
18	34 ± 0.4
19	32 ± 0.2
20	29 ± 0.5
21	27 ± 0.5

**Table 2:** Results of effect of various excipients on gelation temperature.

Concentration of Poloxamer 407 (20% *w*/*v*) + Excipients	Gelation Point in °C ± S.D	Effect on the Gelation Point
Poloxamer 407 + drug (2.5 mg)	27 ± 0.3	Decrease
Poloxamer 407 + Carbopol 934 (0.3% *w*/*v*)	26 ± 0.3	Decrease
Poloxamer 407 + HPMC K4M (0.3% *w*/*v*)	27 ± 0.3	Decrease
Poloxamer 407 + benzyl alkonium chloride (0.01% *w*/*v*)	31 ± 0.3	Increase
Poloxamer 407 + sodium chloride (0.9% *w*/*v*)	32 ± 0.3	Increase

**Table 3:** Result of evaluations performed on factorial batches.

Batches	pH	Viscosity (cps) at 4 °C ± S.D.	Viscosity (cps) at 37 °C ± S.D.	Drug Content (%)	Mucoadhesive Force (Dyne/cm^2^) ± S.D.
PG1	4.95	17 ± 3.3	4965 ± 6.12	95 ± 2.5	1632.09 ± 10.5
PG2	5.15	21 ± 2.45	5123 ± 2.44	94 ± 3.2	2028.3 ± 13.74
PG3	5.10	29 ± 2.36	5441 ± 2.55	92 ± 2.7	2896.8 ± 18.21
PG4	5.18	24 ± 3.21	5785 ± 3.14	95 ± 1.1	3082.09 ± 15.3
PG5	4.99	31 ± 2.6	5320 ± 3.44	96 ± 2.1	2508.3 ± 11.17
PG6	4.79	37 ± 1.0	5031 ± 3.71	93 ± 3.7	2466.8 ± 15.24
PG7	5.22	39 ± 2.5	5392 ± 1.23	94 ± 2.1	2746.5 ± 16.82
PG8	5.09	43 ± 3.1	5782 ± 3.14	91 ± 3.4	3495.5 ± 19.71
PG9	4.82	45 ± 2.28	5938 ± 3.14	90 ± 2.6	3862.5 ± 14.52

**Table 4:** Summary of analysis of variance for response parameters.

Source	Sum of Squares	Degree of Freedom	Mean Square	F Value	*p*-Value
Gelling temperature (°C)					
Model	39.58	5	7.92	57.00	0.0036
Residual	0.42	3	0.14	-	-
Corrected total	40.00	8	-	-	-
Gel strength (s)					
Model	195.11	5	39.02	65.85	0.0029
Residual	1.78	3	0.59	-	-
Corrected total	196.89	8	-	-	-
Drug diffused after 8 h (%)					
Model	74.03	5	15.62	60.26	0.0033
Residual	0.86	3	-	-	-
Corrected total	74.89	8	-	-	-

**Table 5:** Predicted and actual values of the responses for validation run.

Parameter	Gelling Temperature (°C)			Gel Strength (s)			% Drug Diffused (after 8 h)		
Batches	PG10	PG11	PG12	PG10	PG11	PG12	PG10	PG11	PG12
Predicted value	32.21	33.97	35.39	38.41	37.03	33.88	89.91	90.70	92.30
Experimental values	31.25 ± 0.5	32.88 ± 0.8	34.32 ± 0.7	37.24 ± 2.1	36.92 ± 3.21	32.95 ± 2.15	88.78 ± 1.5	89.62 ± 1.3	90.97 ± 0.8
% bias	2.98	3.08	2.77	3.16	2.21	2.74	1.34	1.19	1.44

**Table 6:** Factors along with their examined levels in three-level and two-factor factorial design.

Independent Variables/Levels	Amount of Poloxamer 407	Amount of HPMC K4M
	X_1_ (mg)	X_2_ (mg)
Low	16	0.2
Medium	16.5	0.4
High	17	0.6
Dependent variable	Y_1_ = Gelling temperature (°C) Y_2_ = Gel strength (s) Y_3_ = % Drug diffused (after 8 h)	

**Table 7:** Composition and evaluation of the batches prepared by implementing factorial design.

Runs	Batch Code	Transformed Fractions of Variables *		Gelling Temperature (°C)	Gel Strength (s)	% Drug Diffused (after 8 h)
		X_1_	X_2_			
1	PG1	16	0.2	37 ± 0.32	30 ± 1.1	95.65 ± 0.03
2	PG2	16	0.4	34 ± 0.53	34 ± 3.3	94.12 ± 0.54
3	PG3	16	0.6	31 ± 0.33	39 ± 1.4	91.52 ± 0.01
4	PG4	16.5	0.2	35 ± 0.41	35 ± 4.4	93.65 ± 0.17
5	PG5	16.5	0.4	33 ± 0.32	37 ± 1.3	92.26 ± 0.17
6	PG6	16.5	0.6	30 ± 0.44	42 ± 2.6	89.16 ± 0.02
7	PG7	17	0.2	34 ± 0.28	38 ± 1.1	91.06 ± 0.39
8	PG8	17	0.4	32 ± 0.48	41 ± 3.2	87.52 ± 0.27
9	PG9	17	0.6	31 ± 0.39	47 ± 3.2	85.52 ± 0.39

* Each batch also included 25 mg of pramipexole dihydrochloride, 0.01% *w*/*v* of benzyl alkonium chloride (a preservative), and 0.09% *w*/*v* of sodium chloride (to maintain isotonicity). X_1_ represents the amount of Poloxamer 407 (mg); X_2_ represents the amount of HPMC K4M (mg).

## Data Availability

All data generated and analyzed during the course of this study are included in the article.
